# Case Report: A Cluster of Complications During Carotid Artery Stenting Managed With Peripheral, Coronary, and Imaging Techniques

**DOI:** 10.3389/fcvm.2021.712963

**Published:** 2021-09-09

**Authors:** Piero Montorsi, Stefano Galli, Giovanni Teruzzi, Sarah Troiano, Luigi Caputi, Sebastiano Gili, Daniela Trabattoni

**Affiliations:** ^1^Department of Clinical Sciences and Community Health, University of Milan, Milan, Italy; ^2^Centro Cardiologico Monzino, Istituto Ricovero e Cura a Carattere Scientifico (IRCCS), Milan, Italy; ^3^Neurology Unit, Department of Cerebrovascular Diseases, ASST Hospital of Crema, Crema, Italy

**Keywords:** carotid artery stenosis, carotid artery stenting, intracranial stenting, in-stent restenosis, abrupt vessel closure, imaging technique

## Abstract

We describe the case of a 72-year-old man with severe, asymptomatic in-stent restenosis detected 4 years after index carotid artery stenting (CAS). The patient was deemed at low risk and scheduled for re-angioplasty with a drug-coated balloon as per institution protocol. What at first seemed a simple case suddenly turned into a series of cerebral and vascular complications that were successfully managed with a mix of peripheral, coronary, and imaging techniques.

## Key Clinical Message

Complications during CAS may occur also in patients deemed at low risk. A prompt identification, understanding of mechanism, and treatment are fundamentals steps. This requires dedicated operators with solid background in peripheral and (eventually) coronary interventions and vascular imaging.

## Introduction

Patient risk profile for carotid artery stenting (CAS)-induced cerebral complication is related to clinical, anatomic, and procedural variables. Despite better pre-operative risk assessment and technological advancements, stroke still occurs. Operators should, therefore, be prepared to identify, understand the mechanisms subtended, and choose the most appropriate treatment to manage any type of complication.

## Case Report

**Figure 1:**
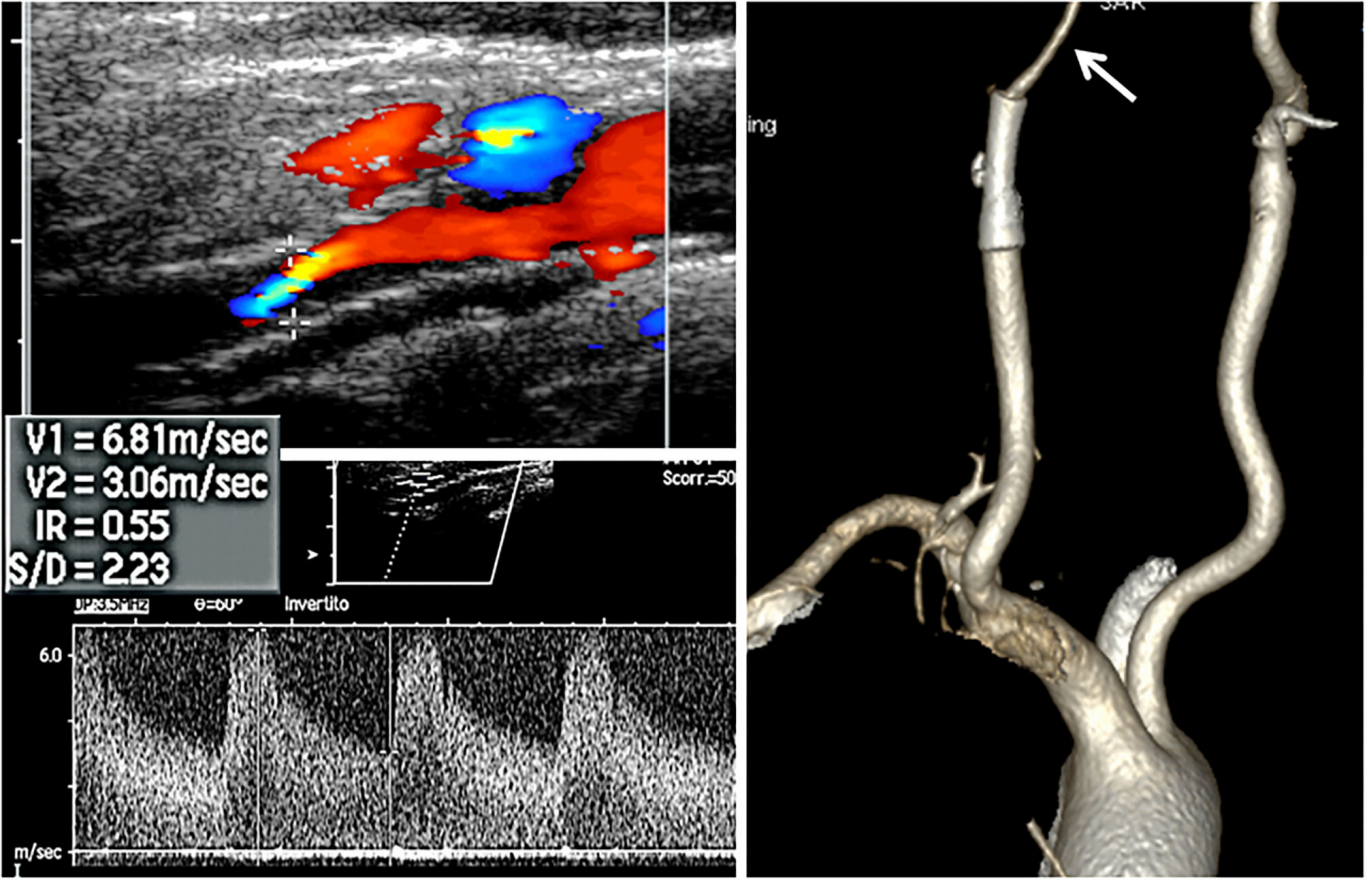
Non-invasive assessment. Doppler ultrasound (US, left side) and computed tomography (CT, right side) volume rendering CT angiography of the patient. Arrow indicates the lumen collapse distal to the stent.

**Figure 2:**
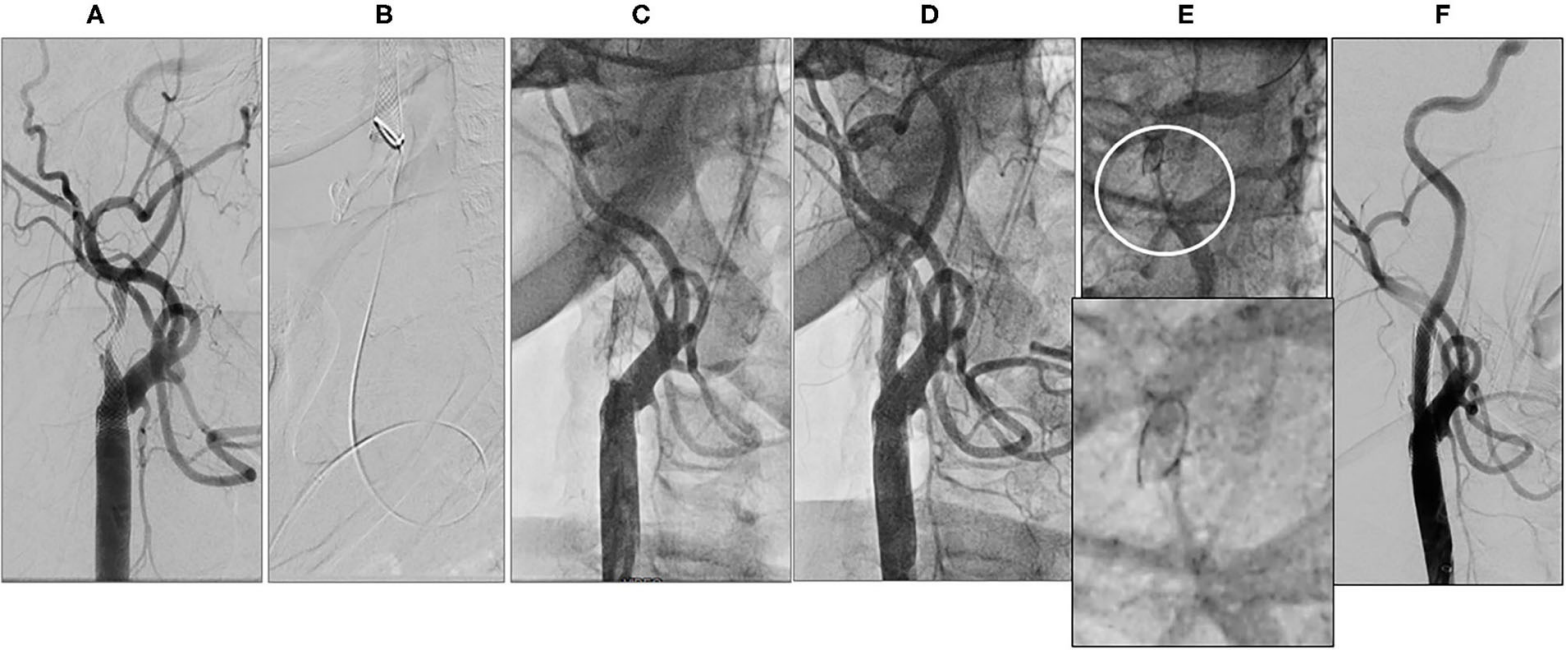
Periprocedural carotid artery stenting step 1. Baseline right internal carotid artery (ICA) carotid in-stent restenosis (CISR) **(A)**. 6F Destination Sheath positioned below the stent **(B)**. Right ICA abrupt occlusion after predilation **(C)**. Right ICA re-opening after aspiration **(D)**. Close up of the Distal ICA (circle) after reopening (**E**, upper part) and close up of filter devise (**E**, lower part). Final digital subtraction angiography (DSA) after drug-coated balloon (DCB) inflation and filter retrieval **(F)**.

**Figure 3:**
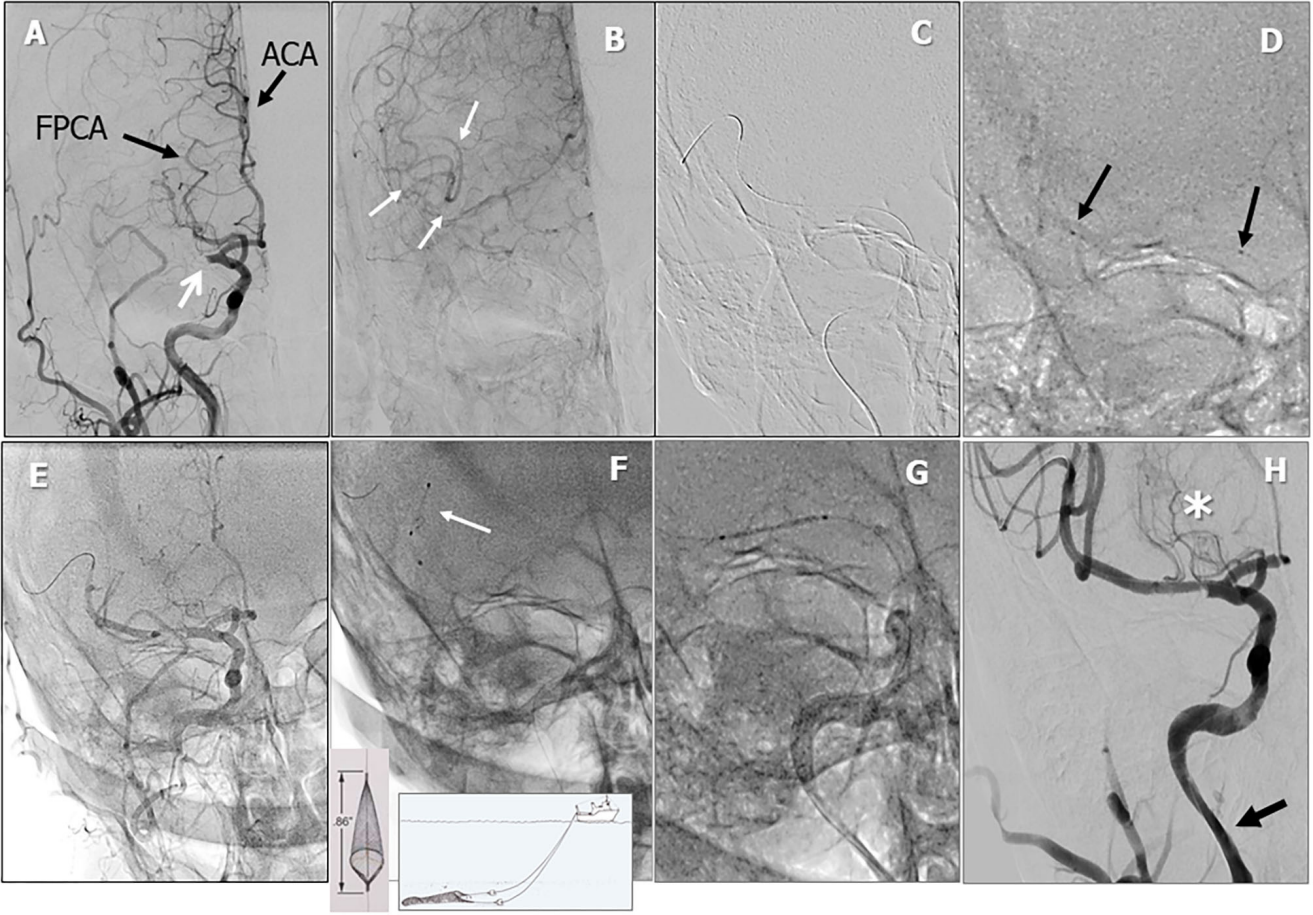
Periprocedural carotid artery stenting step 2. Intracranial PA view showing middle cerebral artery (MCA) tract 1 occlusion (white arrow). Black arrows indicate the fetal posterior multiplanar reconstruction cerebral artery (FPCA) and the anterior cerebral artery (ACA) **(A)**. Leptomeningeal collateral filling of distal MCA tract (white arrows, **B**). Whisper coronary wire (0.014”) in the right MCA **(C)**. Microcatheter (black arrows indicating proximal and distal microcatheter markers) positioned distal to the occlusion and angiographic test **(D)**. Temporary reperfusion after aspiration **(E)**. Failure to retrieve thrombus with a 3-mm Spider Filter **(F)**. Coronary drug-eluting stent (DES) positioning **(G)**. Reperfusion of the right MCA after stent-postdilation (**H**, asterisk: lenticulostriate arteries. Arrow indicates lumen reduction of the distal ICA lumen).

**Figure 4:**
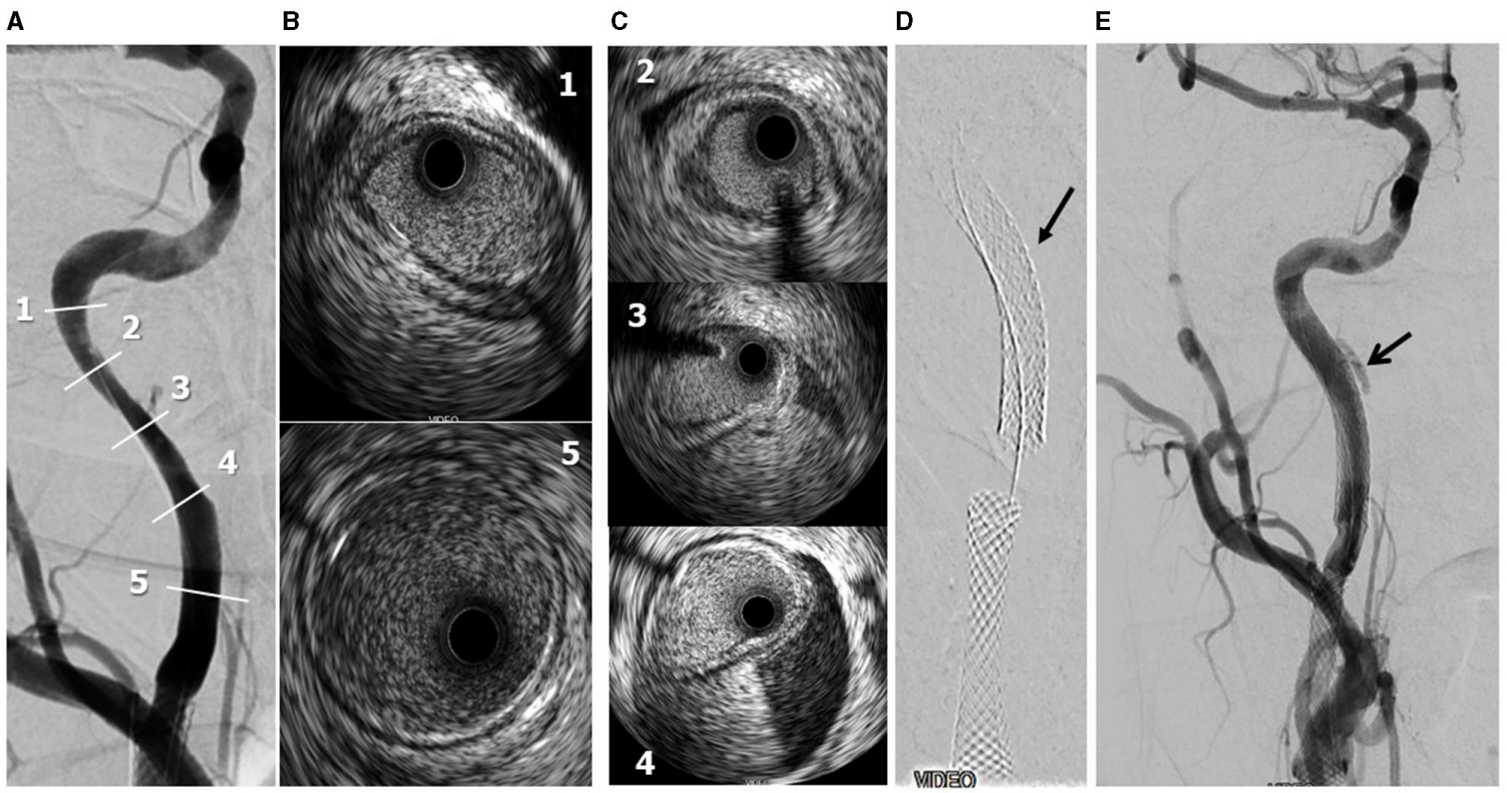
Periprocedural carotid artery stenting step 3. Distal ICA dissection with intramural hematoma and lumen narrowing. Post-reperfusion right ICA angiography. Numbers refer to IVUS images shown in frames **B,C**) **(A)**. IVUS frames 1 and 5 refer to the beginning and end of the intramural hematoma **(B)**. IVUS frames 2-3-4 refer to the true intramural hematoma distribution **(C)**. Following 7 × 20-mm PrecisePro stent positioning (arrow, **D**). Final DSA (arrow: residual intramural hematoma, **E**).

A 72-year-old man was found to have high-grade, asymptomatic carotid in-stent restenosis (CISR, routine) 4 years after right internal carotid artery (ICA) stenting. Previous medical history was unremarkable. Doppler ultrasound (US) showed a peak systolic velocity (PSV) of 6.81 m/s that coped well with in-stent sub-occlusion of the right ICA with distal lumen collapse on computed tomography angiography (CTA) (Figure 1). According to preoperative assessment, the patient was deemed at low risk for complications and scheduled for CAS through a right radial approach. Pre-treatment with double antiplatelet drugs + overnight hydration + i.v. 5,000 U of heparin was done. The right common carotid artery (CCA) was engaged with a 5F Judkins catheter. Baseline digital subtraction angiography (DSA) confirmed a sub-occlusive (95% DS), long CISR (Figure 2A). A 6F 90-cm-long Destination Sheath (Terumo, Tokyo, Japan) loaded on a 0.035” standard wire with a “reshaped tip” was positioned below the stent (Figure 2B). After distal protection device positioning (FilterWire EZ, Boston Scientific, Santa Clara, CA, USA), an intravascular ultrasound (IVUS) run was carried out showing a well-apposed, circular stent with a minimal lumen area of 1.82 mm^2^ (4.6 × 4.7-mm diameter). The plaque showed a heterogeneous aspect with some fragmentations. After predilation with a cutting balloon, 4 × 10 mm, abrupt ICA in-stent occlusion occurred (Figure 2C). Flow restoration was achieved with a 6F Export aspiration catheter passage collecting only white foam. The subsequent DSA imaging showed a wide patent stent with a filling defect close to the filter basket (Figures 2D,E). The patient being asymptomatic, the procedure was completed with a prolonged (3 min) inflation of a 5 × 40-mm In-Pact Admiral Drug-Coated Balloon (DCB; 1:1 stent–balloon ratio at MLA level). Final angiography after filter retrieval (no debris found) showed a good in-stent recanalization result with brisk contrast run-off in both external carotid artery (ECA) and ICA (Figure 2F). Unexpectedly, abrupt occlusion of the M1 tract right of the middle cerebral artery (MCA) was detected. Distal MCA was filled by leptomeningeal collaterals, which were visible on the late arterial frame (Figures 3A,B). The occlusion was quickly crossed with a 0.014” wire loaded into a microcatheter that was seated distal to the occlusion. Having removed the wire, the correct positioning of the wire was checked with contrast injection (Figures 3C,D). Aspiration was attempted with the 6F Export catheter obtaining only transient reperfusion of the distal vessel (Figure 3E). We tried to mechanically retrieve the thrombus with a 3-mm-diameter SpiderRx filter (Medtronic, Santa Rosa, CA, USA) opened distally and pulled back through the occlusion as a “trawl fishing” maneuver (Figure 3F). However, no sustained vessel reperfusion was obtained, and the patient became symptomatic. A Resolute Onyx drug-eluting coronary stent 2.5 × 18 mm (Resolute, Medtronic, Santa Rosa, CA, USA) was positioned and post-dilated allowing brisk distal vessel reperfusion and patient neurological improvement (Figures 3G,H). However, the intracranial PA view showed a smooth lumen reduction of the distal pre-carotid canal ICA segment. A small blood extravasation was also noticed that was thought to be the result of artery dissection fueling an intramural hematoma compressing the ICA lumen. Intravenous nitrate did not change the angiographic appearance. An IVUS run was performed that confirmed this hypothesis and guided the positioning of an additional 7 × 20-mm PrecisePro stent out of the carotid canal (Figures 4A–C) to seal the dissection (Figures 4D,E).

### Post-procedural Phase

The patient was admitted to the cardiac intensive care unit for a few days. A brain CT soon after CAS showed an ischemic lesion of the right basal ganglia that quickly improved over the following 3 days. Doppler US assessment showed a wide patent stent. The patient was discharged on the 10th day on a rehabilitative program.

### Follow-Up

**Figure 5:**
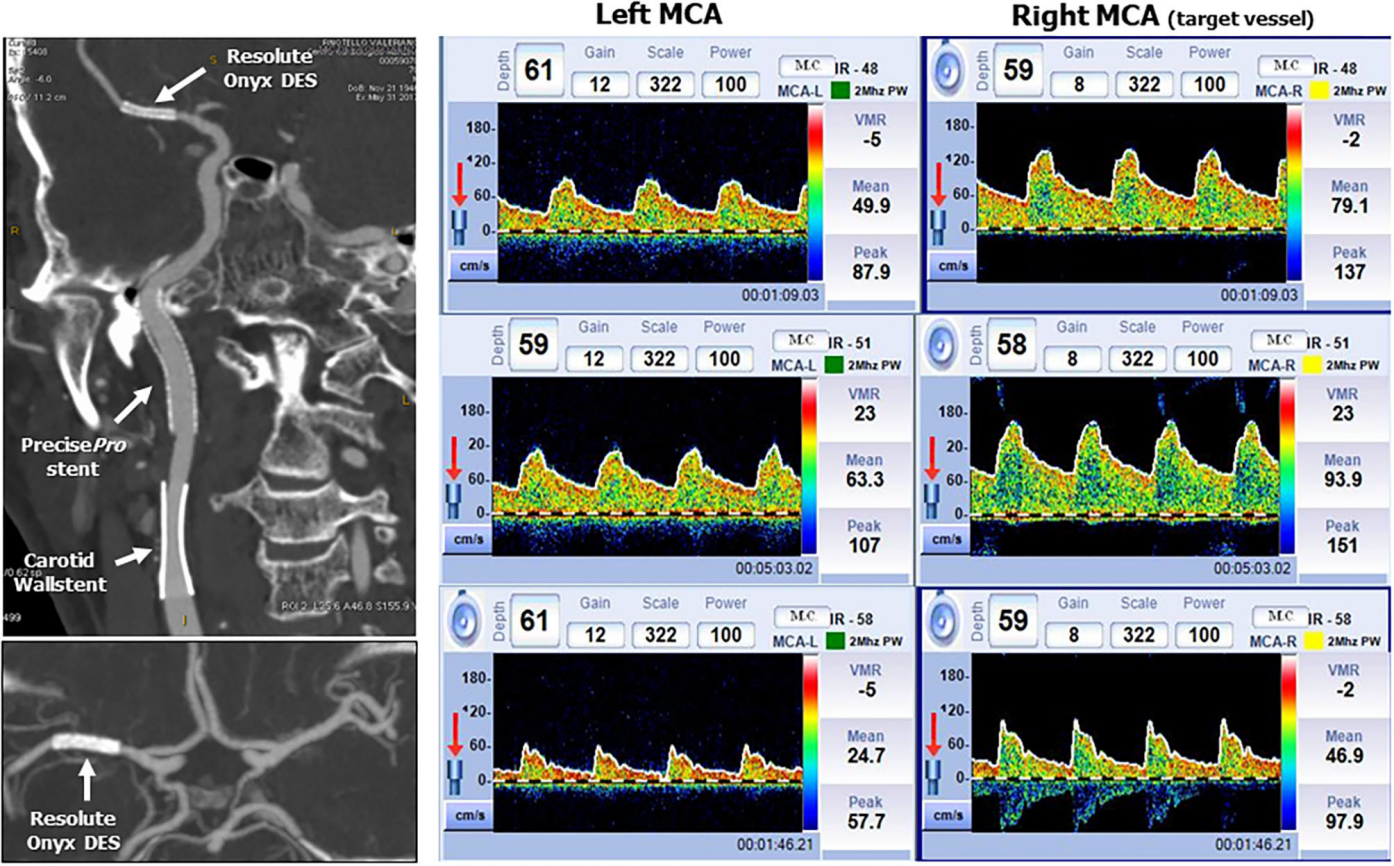
CT angiography and transcranial Doppler at 1-year follow-up. **Left side:** Patency of the three stents by multiplanar reconstruction with maximum intensity projection in the sagittal plane (upper image) and axial plane at the level of Willis circle (lower image). **Right side:** Upper strip: average peak systolic velocity in the left and right (target vessel) MCA. The right MCA PSV is 79 cm/sec indicating a <50% in-stent restenosis. Middle strip: Breath holding test. Left MCA breath holding index 0.82 and right breath holding index 0.75 (normal value >0.69). Lower strip: hyperventilation test: Left MCA average PSV reduction 51%. Right MCA average PSV reduction 40%.

The follow-up was uneventful with no neurological sequelae. Both Doppler US and carotid CT angiography showed wide patency of the three stents at 1-year follow-up (Figure 5). While the patency of both extracranial stents was evident, uncertainty remained for the intracranial stent. We, therefore, used a transcranial Doppler assessment to check the post-stent PSV, which was 79 cm/s indicating <50% restenosis. Moreover, the residual vasoreactivity of both middle cerebral arteries was tested showing a preserved response.

## Discussion

The main take home message from this case report is that CAS-related complications, although rare, may occur either isolated or in a cluster in any patient including those initially deemed to be at low risk (such as those with in-stent restenosis). Operators should be ready to identify them, understand the mechanism(s), and provide an effective treatment that may require techniques (i.e., IVUS), or devices (i.e., coronary stent) not commonly used in carotid interventions. Thus, a wide operator background in peripheral interventions and imaging is mandatory.

Several CAS steps need clarification. First, the high-grade, flow-limiting stenosis of the patient was an indication for revascularization despite lack of symptoms. However, the very late presentation after index CAS (4 years) may raise suspicions of plaque atherosclerotic changes, called “neo-atherosclerosis,” which have been shown to play a role in late stent thrombosis or distal embolization in coronary and carotid arteries (1–3). These changes can be better assessed by imaging techniques, such as IVUS and/or optical coherence tomography (OCT), rather than by standard angiography. The initial IVUS run actually confirmed the severe restenosis pattern with a heterogeneous, fragmented plaque. While no specific strategy has been settled on in these cases, the use of brain protection is highly recommended. Since we systematically use distal filters during CISR treatment, one may wonder if proximal protection (i.e., Mo.MA Ultra system) would be a better choice. However, crossing of the stent struts with the Mo. MA distal channel to enter ECA may be difficult or even unsafe. The alternative is to occlude the CCA with a balloon-tipped catheter (or with the Mo.Ma Ultra mono-balloon system) while maintaining the distal filter. The limitation of this approach is the patient widely patent ECA that would maintain a brisk flow to the target ICA, hampering the protective effect of CCA occlusion (4). Second, target ICA in-stent occlusion after predilation is a rare event. Potential causes include thrombosis, plaque dissection with occlusive flap, or a filter obstructed by embolization of a large amount of debris. While stent thrombosis was excluded by an ACT level >300 s, the site of occlusion (at the proximal stent tract) suggested an occlusive flap. Distal embolization could not be assessed due to the lack of flow. Whatever the cause is, aspiration with a catheter (at least 6F in size) is the first thing to do. The post-aspiration contrast injection was kept long enough to opacify the entire vessel length. A good stent patency was documented with a slow flow and a “minus” defect close to the filter, making distal embolization entrapped into/around the filter basket as the most likely occurrence. Because a further filter aspiration could increase the risk of debris dislodgment, we decided to proceed with DCB inflation. Third, while DCB has been shown to be a valid alternative over POBA or re-stent in CISR (5–7), the limitation is a suboptimal angiographic result requiring “bailout stenting.” Whether a stent placement would have been a better choice for plaque containment in this case is a matter of discussion. However, plaque prolapse has been reported to occur even after CISR re-stenting by OCT (2). Thus, if required, positioning of a double-layer stent might be the right choice (8). In this case, both the angiographic and IVUS final assessment showed a wide patent lumen with brisk blood flow and no dissection allowing distal filter remouval (no debris found inside). Fourth, in the case of large-vessel occlusion—such as MCA M1 tract—mechanical thrombectomy is the recommended reperfusion therapy (9). Unfortunately, no stent retriever was available in our cath lab at that time, and the maneuvers aiming to aspirate/remove the thrombus failed. So, being already in place with the guiding catheter, a third-generation drug-eluting stent (DES) was deployed (as a bailout) with prompt reperfusion and patient neurological improvement. While the use of DES for intracranial atherosclerosis has been reported with good success rates, acceptable complication rates, and minimal ISR rates, no indication exists for periprocedural stroke treatment (10). Fifth, the smooth, tapering reduction of distal ICA diameter with tiny contrast extravasation suggested a dissection with intramural hematoma-induced lumen compression. This complication was likely due to traumatic arterial wall damage during the passage of the several catheters and the open filter retrieval. Imaging with IVUS confirmed the diagnosis, identified the length of the hematoma allowing us to select the proper stent size and length. Stent post-dilation should be avoided for the risk of hematoma shift outside the stent covered tract. Sixth, Doppler US and CT angiography are the best non-invasive methods to check stent patency over time. The long-term patency of the intracranial DES by CT angiography is greatly hampered by the stent “blooming effect” that makes both MLA and stent diameter determination misleading. Transcranial Doppler is a valuable diagnostic tool that may help in these cases by measuring target vessel PSV MCA velocity that is consistent with <50% restenosis (79 cm/s, <100 cm/s ISR <50%) (11). Moreover, no different middle cerebral artery vasomotility to physiologic stimuli was shown on both sides.

## Conclusions

The treatment of extracranial carotid artery stenosis is a complex scenario that may rapidly change into catastrophic complications such as acute ischemic stroke. Each operator should, therefore, be able to diagnose and resolve these complications using coronary, peripheral, and imaging techniques/tools.

## Data Availability Statement

The original contributions generated for the study are included in the article/supplementary material, further inquiries can be directed to the corresponding author/s.

## Ethics Statement

Written informed consent was obtained from the individual(s) for the publication of any potentially identifiable images or data included in this article.

## Author Contributions

All the authors equally contributed to the conception, writing, and discussion of this manuscript.

## Conflict of Interest

The authors declare that the research was conducted in the absence of any commercial or financial relationships that could be construed as a potential conflict of interest.

## Publisher's Note

All claims expressed in this article are solely those of the authors and do not necessarily represent those of their affiliated organizations, or those of the publisher, the editors and the reviewers. Any product that may be evaluated in this article, or claim that may be made by its manufacturer, is not guaranteed or endorsed by the publisher.
